# Spatiotemporal organization and visual experience: a narrative methodological study of song dynasty paintings

**DOI:** 10.3389/fpsyg.2026.1968553

**Published:** 2026-10-21

**Authors:** Min Wen, Xianyu Shao, Xiaojuan Liu

**Affiliations:** 1 College of Liberal Arts, Yangzhou University, Yangzhou, China; 2 School of Fine Arts, Aba Teachers College, Wenchuan, China

**Keywords:** meaning construction, narrative methods, song dynasty painting, spatiotemporal coordination, text analysis, visual narrative

## Abstract

Narrative inquiry progressively breaks beyond the boundaries of linguistic texts, with visual images serving as important vehicles for encoding experience and constructing meaning, thereby making image-based narrative a new frontier in the field of narrative psychology. However, mainstream narrative methods still rely heavily on oral and textual materials, lacking standardized, reproducible qualitative-analytic frameworks for nonlinguistic visual texts such as traditional painting; existing painting studies are often limited to affective descriptions in art history or cultural interpretation, neglecting the psychological structures of the internal organization of visual experience. This study, focusing on Song Dynasty painting, explores the application of narrative analysis to nonlinguistic visual art. By transforming representative paintings and historical documents into structured visual-narrative texts, the study establishes a multidimensional coding system that encompasses spatial hierarchies, narrative nodes, viewing trajectories, and visual expressions. The findings indicate: (1) Song Dynasty painting constructs a stable narrative architecture of “spatiotemporal coordination” through the deep coupling of foreground, middle ground, and negative space; (2) viewing trajectories serve as a crucial structural link between spatial organization and narrative unfolding, allowing static images to be analyzed as continuous visual-narrative structures. (3) Visual-expression strategies such as ink wash and blue-green organize visual focalization, narrative rhythm, and focal rhythm, contributing to the organization of visual experience and the construction of narrative meaning. The study systematically develops a spatiotemporal-coordinated visual-narrative analysis framework tailored to traditional painting, facilitating the transition from aesthetic interpretation of images to standardized narrative-psychology analysis. Consequently, This research expands the applicability of narrative methods to nonlinguistic visual texts and provides traditional Eastern art as a case study for a psychology-oriented study of visual narrative and cross-cultural experience.

## 1 Introduction

Narrative is not only a form of linguistic expression but also a crucial means by which humans organize experience, understand the world, and construct meaning (Gubman and Anufrieva, 2024). Beyond textual narratives, visual narratives likewise bear the functions of encoding experience and generating meaning (Bainbridge et al., 2021). Compared with textual narrative that relies on the progression of linear sequences (Rejwan and Caciularu, 2021), visual narrative (Yang, 2022) constructs implicit narrative logic more through spatial organization (Davidson, 2023), temporal cues (Davidson, 2023), and imagery associations (Chen et al., 2026), making the viewing process an experience that traverses time and space (Yang, 2022). In recent years, research in narrative psychology has progressively breached the boundaries of traditional linguistic texts, beginning to attend to multimodal narratology—images (Li et al., 2019), bodies (Domínguez, 2023), space, and media (Printezis and Koutsabasis, 2026)—and has underscored the significant value of visual texts in experiential representation and meaning construction.

Contemporary visual narratology research has broken away from the single framework of traditional textual narrative, no longer limited to the linear progression of textual exposition (Rejwan and Caciularu, 2021). Instead, it centers on the narratological logic of the visual image itself. Visual narrative, with frame spatial hierarchy (Greenberg, 2021), symbolic imagery (Serafini and Reid, 2024), and viewing rhythm as core carriers, relies on visual syntax and visual symbol systems (Mühlenbeck and Jacobsen, 2020) to translate latent affect (Wang et al., 2026), temporal order, and spiritual connotations into perceptible visual experience (Searls, 2009). By providing visual movement along latent viewing paths and aesthetic perception, it transmits narrative content and extends meaning; it is a nonlinear narrative mode with visual perception at its core, spatial form as its skeleton, and image metaphor as its kernel, and it also establishes a novel research paradigm for the in-depth analysis and aesthetic appreciation of diverse visual arts.

Within this theoretical context, Song dynasty (960–1279) painting, as the exemplary form of Chinese traditional visual art, provides a typical Eastern art exemplar for visual-narrative spatio-temporal construction, imagistic narration, and immersive aesthetic expression (Vinograd, 2019). Distinct from Western linear visual-narrative logic, Song dynasty painting discards overt plot exposition, forming a latent narrative system unique to Chinese aesthetics (Meretoja, 2023). The paintings, relying on landscape layout, journey imagery, negative-space technique, and object pairing, construct multi-layered spatial structures, deftly embedding the temporal dimension within static images and thereby dissolving the dichotomy between time and space (Yin, 2021).

Its pictorial space not only bears the fundamental function of landscape arrangement (Read, 2024), but also prefigures dynamic viewing paths for spectators to wander, look back, pause, and cycle, enabling static imagery to yield a flowing temporal texture and narrative rhythm (Peterson, 2019). This distinctive mode of expression, in which space bears time and viewing generates narrative, constructs a space–time interwoven characteristic of Eastern visual storytelling, provides viewers with potential viewing pathways, and, through progressively enhanced visual perception, guides them into multiple layers of psychological-aesthetic domains (Kenett et al., 2023; Vaisvaser, 2026). This not only foregrounds the Song Dynasty painters’ ascetic, ethereal aesthetic ideals and spiritual pursuits, but also preserves a more complete account of the perceptual paradigms and artistic expressive wisdom embedded in traditional Chinese culture, greatly enriching the theoretical system and aesthetic dimension of visual narrative (Yang, 2022), and constitutes a preserved mode of visual experiential expression in traditional Chinese painting.

At present, research on traditional painting has formed a relatively mature scholarly system, yet evident methodological boundaries persist across disciplines. Existing studies of traditional painting have long centered on image style (Cheng, 2026), brushwork techniques (Cha, 2020), and spatial organization (Davidson, 2023), as well as painter case analyses (Wang et al., 2024), with a focus on the artistic characteristics of Song Dynasty painting, the evolution of schools, and cultural connotations. However, these studies largely remain at the level of image description and formal interpretation, giving insufficient attention to how a continuous narrative structure is generated within the painting. By contrast, art research from a psychological perspective emphasizes the viewer’s emotional experience (Shim et al., 2019), aesthetic preferences, and cognitive processing, typically treating painting as visual material that elicits responses, rather than as a narrative text with a coherent organizing logic for analysis. This conspicuous gap corresponds to the fact that existing visual-narrative research (Cohn and Magliano, 2020) lacks analytic pathways rooted in narrative psychology (Oatley and Djikic, 2018), while psychological studies relatively neglect the internal structural narrative features of visual art.

Current research on Song Dynasty painting’s spatiotemporal expression also exhibits two limitations. First, studies of spatiotemporal relations in traditional painting largely rely on art criticism and aesthetic interpretation (Wang et al., 2024), lacking a structured research paradigm based on narrative analysis and, consequently, a systematic analytic framework that clarifies the relationships among spatial organization, temporal progression, and experiential expression. Second, the methodological path toward “textualizing” painting remains ill-defined; extensive discussions of visual spatiotemporal aspects frequently linger at the level of affective description (Chamberlain et al., 2022; Ciganko, 2016), and there is a lack of operational methods to translate spatial layout (Polkinghorne, 2010), viewing path (Chamberlain et al., 2021), and temporal progression into analyzable narrative units. This impedes the normalization of the interplay among visual elements within a standardized analytical process and restricts deeper dialogue between traditional painting studies and narrative psychology (Oatley and Djikic, 2018).

This study treats Song Dynasty painting as its object of research and, integrating perspectives from visual narrative theory (Cohn and Magliano, 2020), spatial narratology (Nöth, 2020), and narrative psychology (Oatley and Djikic, 2018), attempts to construct a spatiotemporal co-narrative analysis framework oriented toward traditional painting (McGarrigle, 2018; Wallenstein, 2010). The research employs textual-transformation analysis (Brown and Collins, 2021) and narrative-structure decomposition (Lin et al., 2023), converting spatial hierarchies, temporal cues, and imagery symbols within paintings into analyzable narrative units, thereby examining how Song Dynasty painting shapes a continuous representation of psychological experience through spatial organization and temporal arrangement. Specifically, at the level of painting space transformation, guided by the features of Chinese painting’s scatter perspective, the image is decomposed into three hierarchical layers—foreground, middle ground, and background—while distinguishing between the space of tangible objects and the ambience space implied by negative space, thereby transforming static compositional layouts into spatial fields characterized by depth, layering, and order; at the level of viewing paths, relying on the implicit regularities of object arrangement, movement guides, and plot progression, the study traces the complete dynamic trajectory of the observer’s eye movement, image viewing, and scene transitions, transforming passive image viewing into an active, orderly immersive narrative journey and anchoring, for each segment of the viewing path, the corresponding narrative time node and plot content; at the level of media characteristics, focusing on core visual elements such as brushwork texture and color tonality, imagery forms, and the interplay of light and shadow; the study excavates the narrative semantics and meaning construction associated with different visual traits, translating the density of ink and brush, the openness and closure of composition, and the primacy or subordination of objects as the central vehicles for regulating narrative pace, rendering narrative atmosphere, and conveying narrative emotion. The study centers on the following three questions:

RQ1: *How does Song Dynasty painting form a visual narrative structure through spatiotemporal organization?*

RQ2: *What stable narrative patterns do these narrative structures exhibit?*

RQ3: *How do these narrative patterns organize visual experience and meaning construction?*

Through the study of the above content, the contributions of this research are twofold. (1) It attempts to transform Song Dynasty painting into a structured visual-narrative text by extracting spatial nodes, temporal cues, and visual imagery, thereby bridging the boundary between traditional image appreciation and textual analysis, and integrating static paintings into a narrative-analytic framework to provide a workable, textualized pathway for visual arts research. (2) It proposes the “spatiotemporal co-narrative” analysis framework, foregrounding the connections among spatial organization, viewing paths, temporal progression, and psychological experience, and revealing how traditional painting expresses experience and constructs meaning through spatial organization. This approach not only broadens the application of narrative psychology within the visual arts but also offers experiential materials and analytical perspectives from Eastern traditional art to cross-cultural visual-narrative research.

## 2 Literature review and theoretical framework

### 2.1 Visual narrative and meaning construction

Narratives play a crucial role in understanding and constructing meaning about the world (Yang, 2023). Bruner contends that humans organize discrete experiences into a continuous, meaningful whole through narrative, making narrative an essential cognitive mode for understanding experience rather than merely a tool for information transmission (Bruner, 1986). Polkinghorne further argues that the core function of narrative is to integrate time, events, and actions into an inherently interconnected experiential structure, enabling individuals to comprehend their relationship with the world across the continuity of experience (Polkinghorne, 2010). In recent years, as narrative inquiry has continually transcended the boundaries of linguistic texts (Sadirova and Sydyk, 2024), multimodal expressions such as images (Cheng, 2026), space (Greenberg, 2021), embodiment (Domínguez, 2023), and digital media (Lesage et al., 2025) have increasingly become important objects of narrative research; visual narrative is emerging as a pivotal area of cross-disciplinary attention between narrative studies and psychology (Oatley and Djikic, 2018).

Unlike word-based narratives that advance through linguistic sequence, visual narratives (Cohn and Magliano, 2020) rely primarily on spatial organization (Tyler, 2007), viewing paths (Beelders and Bergh, 2020), visual focal points, and temporal cues to construct narrative logic in a coordinated manner. The spatial relations within images not only perform compositional functions but also function as important media for narrative progression (Intxaurbe et al., 2024); viewers, during the act of viewing, continually move their gaze, discern spatial hierarchies, and establish visual associations, thereby forming a continuous process of experiential understanding (Ji et al., 2025). Accordingly, visual images not only present information but, through the coordinated organization of space and time, realize meaning construction. In recent years, visual narrative has been widely applied across fields such as film, photography, and digital media; however, narrative research on traditional painting—a quintessential visual text—remains relatively underdeveloped.

### 2.2 Narrative methods and visual text analysis

As narrative research shifts from theoretical discussion to methodological practice, narrative analysis has become an important research method in psychology (Oatley and Djikic, 2018; Yang, 2023), anthropology (Ramchandani, 2016), education (Pires Pereira and Campos, 2023), and cultural studies (Piipponen and Karlsson, 2021). Riessman contends that narrative analysis attends not only to narrative content but also to narrative structure, modes of organization, and processes of meaning generation; the focus extends from “what is told” to “how experiences are organized” (Riessman, 2008). Herman proposes that narrative exists not only in linguistic texts but in images, space, and visual media; any expression that possesses temporal organization and event-relatedness can be understood as a narrative text (Yang, 2022). In this study, the “visual text” is treated as the primary object of narrative analysis—the original painting images themselves—not materials that have been transformed into linguistic text. The study further undertakes the “textualization” of the visual text, in accordance with a predefined coding framework, translating the image’s spatial hierarchy, temporal cues, narrative nodes, viewing paths, and visual expressions into structured units of analysis. Accordingly, the visual text is the object of study, textualization coding constitutes the analytic process, and the two should not be equated.

In the field of visual studies, narrative methods have gradually evolved from traditional image interpretation to structured analytical pathways (Yang, 2022). Researchers have begun coding visual texts by identifying visual units, viewing paths (Chamberlain et al., 2021), narrative nodes, and spatial transitions, thereby endowing images with an analytical structure similar to linguistic texts. This approach not only enhances the transparency and reproducibility of visual research but also propels visual arts scholarship from affective interpretation to standardized analysis. Existing narrative methods have been predominantly applied to modern imagery, photography, advertising design, and digital media (Yang, 2023); for traditional painting—especially Chinese classical painting—there remains a lack of mature methodological frameworks for visual narrative analysis. How to transform static paintings into analytically meaningful visual narrative texts and to establish standardized coding schemes remains a critical direction for the development of narrative-method research.

### 2.3 Main advances in song dynasty painting research

As an important stage in the development of traditional Chinese painting, Song Dynasty painting has long been a major object of study in traditional painting research. Current research has formed three principal directions. First, studies on form language and spatial organization. These studies focus on pictorial composition, revealing differences between Northern Song Dynasty (960–1127) panoramic composition and Southern Song Dynasty (1127–1279) corner-style composition, and interpret the aesthetic features embodied by different compositional modes from the perspective of the evolution of artistic style (Chamberlain et al., 2022). Second, research on traditional visual narrative and cultural context. This work centers on artist case studies, school evolution, literati culture, and the historical background, analyzing the development process and cultural connotations of Song Dynasty painting through documentary evidence to provide a rich historical basis for understanding Song Dynasty painting (Yang, 2022). Third, studies on image symbols and themes. Researchers analyze images such as landscapes, figures, travel, and recluse figures, exploring the cultural meanings and artistic expressions carried by different visual symbols, and conducting relatively in-depth case studies of works across different subjects (Larrea Solórzano et al., 2023). Existing research reveals Song Dynasty painting’s artistic style, cultural background, and image features; most studies remain at the level of image form, aesthetic significance, and cultural interpretation, with limited attempts to treat painting as a continuous, organized visual narrative text (Cohn and Magliano, 2020). How spatial relations within the image (Intxaurbe et al., 2024), viewing paths, temporal cues, and narrative structure jointly organize the visual experience still lacks a systematic analytical framework.

### 2.4 Research gaps, positioning, theoretical framework

In sum, at present, visual-narrative research (Cohn and Magliano, 2020) and Song dynasty painting studies have developed relatively mature trajectories, yet there remains an evident methodological discontinuity between them. On one hand, narrative psychology and visual-narrative studies emphasize experience organization, meaning construction, and analysis of narrative structure, yielding comparatively mature theories of narrative analysis; however, related work is largely concentrated on linguistic texts (Sadirova and Sydyk, 2024), digital media (Lesage et al., 2025), and contemporary visual materials, lacking an adaptable analytic pathway for traditional painting. On the other hand, existing narrative studies have deeply illuminated the formal features, cultural value, and iconography of Song dynasty painting, yet seldom employ narrative-analytic methods to systematically encode the painting’s spatial relations (Intxaurbe et al., 2024), viewing paths (Chamberlain et al., 2021), and temporal structure, leaving the visual-textual attributes of traditional painting underexploited. Accordingly, at least three methodological problems exist: (1) a lack of standardized analytic pathways to translate traditional painting into a visual-narrative text; (2) a lack of a spatiotemporal, coordinated narrative framework capable of integrating spatial organization, temporal progression, and viewing path; (3) a lack of empirical studies based on narrative-analytic methods that discuss how traditional painting organizes visual experience and achieves meaning construction. To address these shortcomings, this article selects Song dynasty painting as the object of study and, building on narrative psychology (Oatley and Djikic, 2018; Yang, 2023) and visual-narrative studies (Cohn and Magliano, 2020), adopts a narrative-text analysis method (Yang, 2022) to translate spatial hierarchies, temporal cues, narrative nodes, and viewing paths in paintings into analyzable narrative units. Thereby constructing a spatiotemporal co-narrative analysis framework oriented to traditional painting (Figure 1). The study aims to provide Chinese classical painting with a visual-narrative analytic pathway that is transparent, operable, and reproducible, while further expanding the application of narrative methods in traditional visual-arts research and offering experiential material and new analytic perspectives from Eastern traditional art to cross-cultural visual-narrative research. However, this study does not treat spatial organization, temporal cues, and visual-media characteristics as independent analytic variables. Instead, it conceptualizes them as interdependent structural dimensions of visual narration. Spatial hierarchy provides the structural field within which the narrative unfolds; temporal cues and viewing trajectories endow spatial relations with sequentiality and continuity, while brushwork, coloring, and the treatment of the virtual and the real further modulate visual focus, rhythm, and scene transitions. Collectively, in the course of a shared narrative organization, the three elements realize spatiotemporal coordination, enabling a static image to exhibit an analyzable, continuous narrative structure.

**Figure 1:**
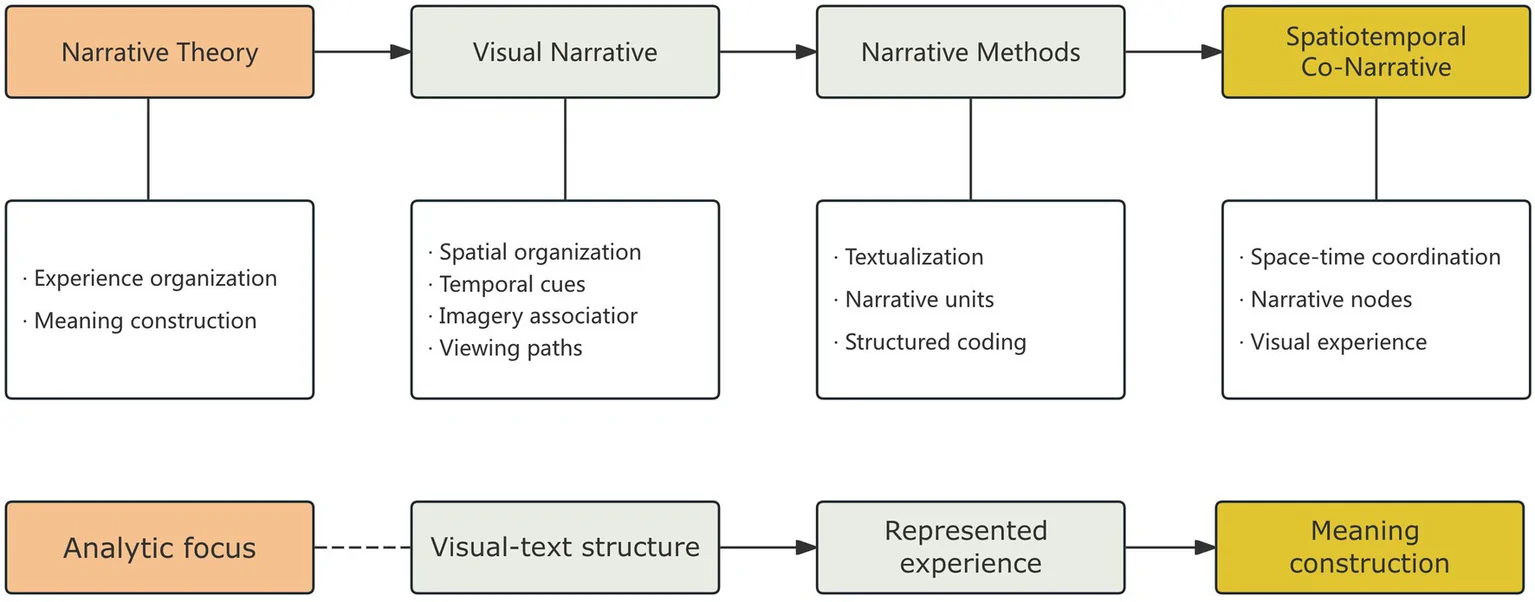
Theoretical framework. The framework integrates narrative theory, visual narrative, and narrative methods to conceptualize Song Dynasty painting as a structured visual-narrative text. It develops a spatiotemporal co-narrative framework integrating spatial organization, temporal cues, narrative nodes, and viewing paths to examine how visual texts organize represented experience and construct meaning. The framework does not infer the actual psychological states of historical painters or viewers.

## 3 Methods

### 3.1 Research design

This study adopts qualitative textual analysis (Brown and Collins, 2021), with “spatiotemporal coordination” and “visual experience” as the core analytical perspectives, to investigate the narrative structures in Song Dynasty painting. Unlike traditional painting studies that emphasize stylistic classification and formal feature analysis, this paper focuses more on how internal organization within paintings—through spatial hierarchies, viewing paths (Chamberlain et al., 2021), and temporal progression—produces a continuous narrative organization. From a methodological standpoint, this article analyzes Song dynasty painting as a visual narrative text (Cohn and Magliano, 2020). Here, the term “visual text” refers to the original painting images that possess an internal organizational structure, visual cues, and meaning associations; the “textualization” in the study does not reduce the image to a linguistic text, but rather, through structured coding, transforms visual relations into analyzable narrative units. In the study, elements such as the painting’s landscape structure, the actions of figures, spatial transitions, visual whitespace, and viewing sequence are converted into analyzable narrative units, thereby discussing how the visual image organizes the viewing experience and the expression of meaning (Lee and Lee, 2026). This study focuses on the structural features of the visual narrative text, not on assessing the artwork’s artistic value—particularly the internal spatiotemporal organization and its meaning-construction process within the visual narrative text—and not on inferring the historical painter’s genuine psychological state or the observer’s actual mental responses. The study’s conclusions are limited to the textual-analytic level of the visual-narrative structure and do not involve inferences about psychological mechanisms or causal relationships. Likewise, the viewing path in this study is an analytically reconstructed path based on the visual structure. Rather than an actual viewing trajectory obtained from eye-tracking data; visual experience refers to the forms of experience represented and organized by the artwork, rather than direct measurement of the real observer’s subjective experience.

### 3.2 Research corpus and selection criteria

The materials analyzed in this study consist of two parts. Representative Song Dynasty (960–1279) paintings, and related colophons, painting treatises, and historical textual sources. Following Wallenstein’s study of space and time in Laocoön (Wallenstein, 2010) and McGarrigle’s work on artistic narration (McGarrigle, 2018), this study defines the material selection criteria as narrative integrity and spatiotemporal expressive features.

The specific inclusion criteria include: (1) The work must be attributed to Song Dynasty painters whose authorship is clearly documented in historical sources, including representative painters from both the Northern Song and Southern Song periods. (2) The works must exhibit relatively clear spatial organizational structures and viewing paths, including landscape handscrolls, travelogues, multi-layered landscape structures, or other visual texts with spatiotemporal unfolding features. (3) The sample works must be relatively well-preserved and have clear scholarly corroboration to ensure the authenticity of the objects and textual stability. (4) The works must have corresponding ancillary textual materials, including colophons, painting treatises, or related documentary records, to support interpretation of the pictorial narrative context. This study selects 22 Song Dynasty paintings that meet the inclusion criteria and exhibit spatiotemporal organizational features as the objects of analysis, and, together with documentary texts, constructs a research corpus to cover the major narrative types and spatial expressions in Song Dynasty painting.

### 3.3 Analytical framework and coding system

This study integrates Visual Narrative Theory (Cohn and Magliano, 2020; Yang, 2022), Spatial Narrative Studies (Zhao and Zhang, 2024), and Chinese traditional painting theory to construct a dual-layer analytical framework comprising visual structure and narrative-path analysis, and to examine the research corpus using a hierarchical coding scheme (Lee and Ha, 2026). At the level of visual-structure analysis, the focus is on identifying spatial organization and temporal progression within the imagery, encompassing three dimensions: (1) Coding of spatial hierarchical construction, including near/far stratification, path transitions, and the use of negative space as visual expression; (2) Coding of viewing-path drift (Khatib and Potash, 2021), including character actions, spatial turns, visual focal points, and contextual transitions; (3) Coding of medium characteristics, principally around coloring techniques, ink-tonality gradation, and brushwork rendering. (4) Cross-dimensional collaborative encoding, primarily identifying the co-occurrence relationships among spatial hierarchy, temporal cues, narrative nodes, viewing paths, and the manifestations of the visual media; with particular emphasis on recording which spatial nodes initiate the visual focal point, how viewing paths transform, and how brushwork, color design, and the treatment of the real and the illusory synchronize with spatial transformations and narrative nodes.

In narrative experience analysis terms, the study further attends to the organizational modes of experience formed during viewing, including visual movement paths, viewing rhythm, spatial immersion, and contextual continuity, to discuss how the image achieves a continuous visual experience through spatiotemporal organization. In this study, “viewing path” is not an empirical measurement of actual viewer eye-tracking trajectories, but rather a textual reconstruction of the potential viewing sequence offered by the work, based on the visual focal points within the frame, connections between objects, spatial inflections, the actions of figures, and cues derived from deliberate negative space. Accordingly, this metric reflects the narrative path presented by the visual text, rather than the actual eye movements of viewers. All coding dimensions were repeatedly revised and inductively consolidated during the pre-analysis phase to enhance the stability and applicability of the analytic framework. Throughout the study, standardized coding logic was uniformly employed to reduce subjective bias in the interpretation process.

### 3.4 Data analysis

This study adopts a three-stage analysis workflow—“Material Organization-Open Coding-Narrative Induction”—and employs an iterative analysis approach to continually revise and refine the analytic framework (Carson and Strakoš, 2020). Stage 1: Material Organization. Paintings and related textual materials are uniformly coded and archived, with systematic documentation of epoch, author background, subject type, and textual sources, establishing a standardized research corpus. Stage 2: Open Coding Analysis. Guided by the established coding framework, visual texts and related literature are analyzed item by item to identify spatial structures, temporal progression modes, and narrative nodes, yielding initial conceptual labels. Subsequently, related concepts are clustered to progressively form stable narrative categories. Stage 3: Narrative Induction and Contextual Interpretation. Drawing on Song Dynasty history and culture, landscape aesthetics traditions, and modes of visual perception, coding results are interpreted further to analyze the relationships between spatiotemporal organization and visual experience in Song Dynasty painting and to synthesize the principal narrative patterns. Stage 4: Cross-dimensional Co-coding. After completing the initial coding of spatial hierarchies, temporal cues, narrative nodes, viewing trajectories, and media characteristics, perform a further comparison of co-occurrence, coherence, and transformation relationships across the coding dimensions, with a focus on identifying how spatial transformations correspond to changes in viewing trajectories, how viewing trajectories connect narrative nodes, and how media manifestations synchronize with spatial and trajectory changes.

### 3.5 Trustworthiness and validity controls

To enhance the credibility of findings and analytic consistency, this study employs multiple quality-control strategies (Susman-Stillman et al., 2018). In terms of material credibility for inclusion, all study samples originate from authoritative collections, formally published catalogues, and core scholarly literature to ensure the authenticity and stability of the objects under study. Coding is conducted independently by the researchers, followed by sampling cross-checks by two peer scholars with backgrounds in visual arts and narrative psychology; for any aspects of spatial nodes and viewing-path determinations with divergences, iterative discussion is conducted until consensus is reached. Additionally, the study maintains complete coding records, textual annotations, and analytic memos to enhance transparency and traceability of the research process.

### 3.6 Researcher reflexivity

Because this study employs qualitative text analysis, researchers inevitably participate in meaning-making during text identification, narrative-unit segmentation, and contextual interpretation. Accordingly, the study places particular emphasis on reflexive control in the research process to mitigate the influence of the researcher’s preconceptions on text interpretation (Bourke, 2014). In the course of the study, researchers consistently regard Song Dynasty paintings as visual narrative texts endowed with historical context and cultural particularities, rather than as artifacts for theoretical application through a contemporary psychology framework. To avoid a bias toward subjective interpretation, the research process continuously employs iterative comparison among text, context, and theory, revising and calibrating initial coding results, and cross-validating them with Song Dynasty painting theory, painting inscriptions and colophons, and related historical sources to strengthen the contextual coherence of narrative interpretation. This study did not adopt eye-tracking experiments or reader-response experiments, because the aim is to establish a narrative-analytic pathway for visual texts rather than to measure actual observers’ eye movements or psychological responses. Consequently, the viewing path described in this article constitutes an analytic reconstruction based on visual structure, its validity primarily supported by textual evidence and coding consistency. Future research could further integrate eye-tracking, process-tracing of viewing, or reader-response experiments to empirically validate the viewing path proposed herein. Moreover, the research process preserves complete analytic memos and coding revision records to illustrate how the researcher progressively forms narrative generalizations and theoretical explanations from the visual text, thereby enhancing the transparency and traceability of the research process.

### 3.7 Ethical statement

This study is a narrative analysis based on historical visual texts and publicly available documentary materials. And does not involve recruitment of human subjects, experimental interventions, collection of personal privacy data, or biomedical research content, and therefore does not require ethical committee approval. The paintings used, colophons, and historical documents originate from publicly published catalogues of institutional holdings, formal bibliographic works, and scholarly research materials. Throughout the study, strict adherence to academic norms and citation principles was maintained to ensure legitimate use of materials and scholarly integrity.

## 4 Results

### 4.1 Overview of the selected works

Based on the inclusion criteria, this study ultimately selected 22 representative Song Dynasty paintings as the core analytical sample (Table 1), and, in conjunction with related inscriptions, painting treatises, and historical literature, constructed an auxiliary textual corpus. The sample spans the two historical periods of the Northern Song and the Southern Song, comprising five Northern Song painters: Cheng Li (3 works), Kuan Fan (2 works), Xi Guo (1 work), Ximeng Wang (1 work), and Youren Mi (1 work); and four Southern Song painters: Yuan Ma (7 works), Tang Li (4 works), Gui Xia (2 works), and Boju Zhao (1 work).

**Table 1:** Classification statistics of included works.

Subject	Artist	Period	Quantity	Representative works (Chinese)	Representative works (Chinese Pinyin and English)	Code
Landscape painting	Cheng Li	Northern Song Dynasty (960–1127)	3	《群峰雪霁图》	Qunfeng Xueji Tu (Snow-Capped Peaks After Snow)	A01
				《晴峦萧寺图》	Qingluan Xiaosi Tu (Clear Peaks and a Desolate Temple)	A03
				《寒林骑驴图》	Hanlin Qilü Tu (Riding a Donkey in a Winter Forest)	B06
Landscape painting	Kuan Fan		2	《雪景寒林图》	Xuejing Hanlin Tu (Snow Scene in a Winter Forest)	A05
				《溪山行旅图》	Xishan Xinglü Tu (Travelers Among Mountains and Streams)	B07
Landscape painting	Xi Guo		1	《早春图》	Zaochun Tu (Early Spring)	A02
Landscape painting (blue-and-green)	Ximeng Wang		1	《千里江山图》	Qianli Jiangshan Tu (A Panorama of Rivers and Mountains)	C03
Landscape painting	Youren Mi		1	《潇湘奇观图》	Xiaoxiang Qiguan Tu (Wondrous Views of the Xiaoxiang Region)	C01
Landscape and figure painting	Yuan Ma	Southern Song Dynasty (1127–1279)	7	《梅石溪凫图》	Meishi Xifu Tu (Plums, Rocks, Stream and Ducks)	A07
				《秋江渔隐图》	Qiujiang Yuyin Tu (Fisherman in Reclusion on an Autumn River)	B03
				《松下闲吟图》	Songxia Xianyin Tu (Leisurely Chanting under Pines)	B05
				《踏歌图》	Tage Tu (Dancing and Singing)	B08
				《雪羁观梅图》	Xueji Guanmei Tu (Viewing Plums in Snow)	B09
				《华灯侍宴图》	Huadeng Shiyan Tu (Banquet by Lamplight)	B10
				《倚松图》	Yi Song Tu (Leaning on a Pine)	C02
Landscape and figure painting	Tang Li		4	《万壑松风图》	Wanhe Songfeng Tu (Windy Pines Among a Myriad Valleys)	A04
				《采薇图》	Caiwei Tu (Gathering Edible Herbs)	B01
				《晋文剬复国图》	Jin Wengong Fuguo Tu (Duke Wen of Jin Restores His Kingdom)	B02
				《濠梁秋水图》	Haoliang Qiushui Tu (Autumn Water at Haoliang)	B04
Landscape painting	Gui Xia		2	《溪口垂钓图》	Xikou Chuidiao Tu (Fishing by a Stream)	A06
				《溪山清远图》	Xishan Qingyuan Tu (Pure and Remote Mountains and Streams)	A08
Landscape painting (blue-and-green)	Boju Zhao		1	《江山秋色图》	Jiangshan Qiuse Tu (Autumn Colors of Rivers and Mountains)	C04

Based on the distribution of subjects among the selected works, the corpus is dominated by landscape painting, while also incorporating scenes of human activity and narrative imagery, reflecting experiential organization within a specific historical-cultural context. Together, these samples provide a relatively comprehensive reflection of visual features in Song Dynasty painting, including spatial hierarchy, viewing pathways, and scene construction. In expressive form, the corpus encompasses both the ink-wash and blue-green coloration systems, covering representative modes of visual expression in Song Dynasty painting and offering an analytical basis for comparing narrative structures across different visual languages.

From composition features, Northern Song works present panoramic, multi-layered, progressive spatial structures, emphasizing depth and overall layout; Southern Song works more often employ corner-type compositions with deliberate use of negative space, highlighting local scenes and visual focal points. The samples from different historical periods exhibit clear differences in spatial organization, viewing pathways, arrangement, and narrative presentation, providing rich material for subsequent identification of spatiotemporal co-narrative modes in Song Dynasty painting.

Overall, the selected sample covers the core developmental stages of Song Dynasty painting, representative painters, mainstream thematic subjects, and typical spatial organizational forms and visual expressions. The samples display strong representativeness, variability, and completeness, effectively supporting this study’s systematic deconstruction and synthesis of the spatial organization logic, viewing-narrative mechanisms, construction of visual experience, and spatiotemporal co-narrative modes in Song Dynasty painting.

### 4.2 Encoding analysis and thematic extraction

Based on the aforementioned subject features, differences in spatial organization, and types of visual language in the samples, this study employs text-analysis methods to perform per-work, hierarchical, systematic visual deconstruction and narrative decoding for 22 sample works (Table 2).

**Table 2:** Analysis of coding of included works.

Theme	Representative work number	Theme extraction	Section
1	A01, A02, A03, A04, A05, A06, A07, A08	Narrative construction through spatial hierarchy	4.3.1
2	B01, B02, B03, B04, B05, B06, B07, B08, B09, B10	Dynamic visual narrative through shifting viewing paths	4.3.2
3	C01, C02, C03, C04	Construction of narrative meaning through media characteristics	4.3.3

First, the sample encompasses two typologies: panoramic Northern Song spatial forms (A01–A05) and corner-focused Southern Song spatial forms (A06–A08). Building on differences in spatial organization and spatial morphology, we conduct a spatial-hierarchical construction coding study, coding for the arrangement of depicted objects, spatial hierarchy, the form–void relationship, and modes of scene extension; by stripping away superficial stylistic differences between the Northern and Southern Song periods, we summarize the common spatial narrative rules of Song painting.

Secondly, among all sample works, excluding purely landscape pieces, there are paintings in which landscapes coexist with Figures (B01–B10). These works all exhibit a clear visual center of gravity and viewing cues, whether through the depth extension of panoramic landscapes (B06–B10) or the focused composition at the corners (B01–B05), thereby presetting the viewer’s visual flow. Based on the arrangement of sample scenes and features of visual guidance, we encode the trajectories of viewing paths. This study reconstructs the latent viewing paths offered by the compositions by encoding the position of visual focal points, the logic of object linkage, the rhythm of spatial transitions, and the guidance provided by negative space, and derives the narrative regularities by which static images, anchored to visual trajectories, generate dynamic temporal sequences.

Finally, the sample set comprehensively covers the two Song-era visual expression systems, ink wash (C01) and blue–green (C02–C04). These two visual languages shape distinct visual textures through differential ink tonal values, brushstroke cadence, layering of color, and chromatic order. Based on the samples’ ink and color characteristics, we perform medium-specific encoding. The study systematically codes the ink–tone levels, the form–void handling, the color-design logic, and the visual atmosphere; it reveals that different visual expressions are not independent aesthetic forms, but all serve to optimize spatial order and regulate viewing rhythm, ultimately contributing to the selection of narrative information, organization of visual experience, and construction of meaning.

In sum, grounded in the samples’ diverse image features and informed by Song painting conventions and the study’s core objectives, this research establishes an encoding framework along three dimensions—spatial structure, narrative pathway, and visual medium expression. By coding the works, it achieves an inductive progression from concrete image features to narrative logic (Table 3).

**Table 3:** Dimensional and operational indicators.

Analytical dimensions	Operational criteria	Encoding focal points	Representative
Spatial hierarchy	Foreground/middle ground/background; physical space; negative space; spatial transformation	Depth, hierarchy, emptiness/solid, open/closed	A05, A06, A07, A08
Temporal cues	Scene sequencing; continuity of action; repetition/juxtaposition of imagery; visual rhythm	Sequence, extension, pause, and recurrence	A01, A02, A03, A04
Narrative nodes	Entry point; visual focal point; figure action; scene transition; convergence point	Positioning of events/situations	B01, B03, B04
Viewing path	Entry—stay—transition—extension—retrospection/closure	Pathways linking visual focal points	B02, B05, B06, B07, B08, B09, B10
Media representation	Ink tone variation; brushstroke rhythm; color hierarchy; figure–ground contrast	Foci, rhythm, atmosphere, and spatial boundaries	C01, C02, C03, C04
Cross-dimensional coordination	Space × time; space × path; path × medium; the three co-occur	Narrative organizational relationships	A03, B06, B07, C02

### 4.3 Core findings of the encoding of spatiotemporal co-narratives

Based on the framework of visual-structure encoding and narrative-path encoding, this study conducts a systematic survey and analysis of Song dynasty painting samples. The overall encoding results indicate that the existing spatiotemporal co-narrative system of painting is built around three core dimensions: spatial hierarchical structure, continuous viewing paths, and medium characteristics. The three elements are interlinked and mutually reinforcing, transforming static visual forms into a dynamic, orderly visual narrative system with complete expressive function, thereby constituting the implicit narrative-generating logic of Song dynasty painting.

#### 4.3.1 Spatial hierarchy: a hierarchical construction of the narrative structure

The results of the spatial-hierarchical construction encoding show that the picture space of a painting is not merely a backdrop for scenery but a structural framework bearing core narrative functions (Figures 2, 3). The works establish a normative visual narrative order through a three-tier spatial division—foreground, middleground, and background—where different space levels carry differentiated narrative functions, collectively enabling the layered unfolding of narrative content and the logical construction of the narrative.

**Figure 2:**
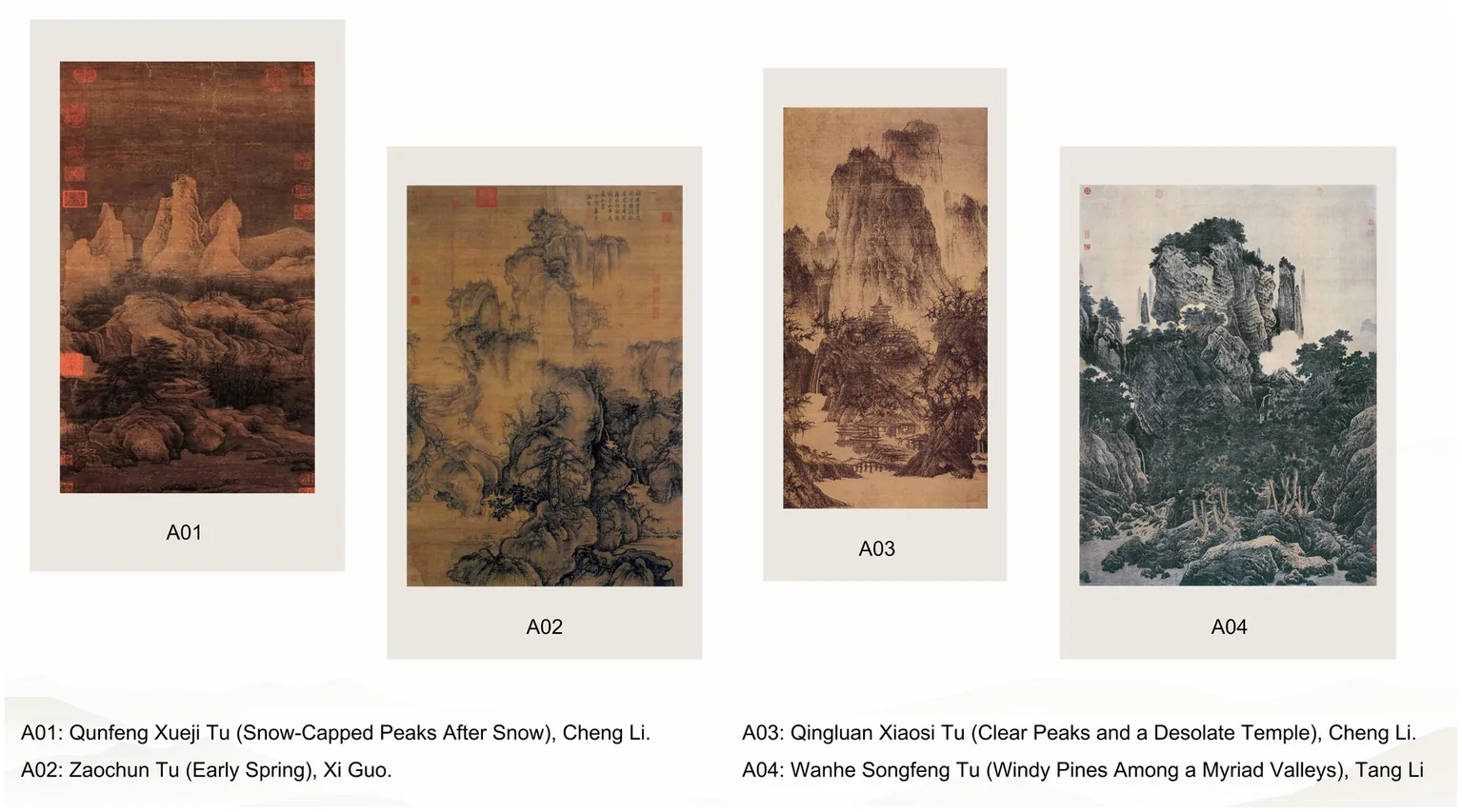
Spatial layering of works.

**Figure 3:**
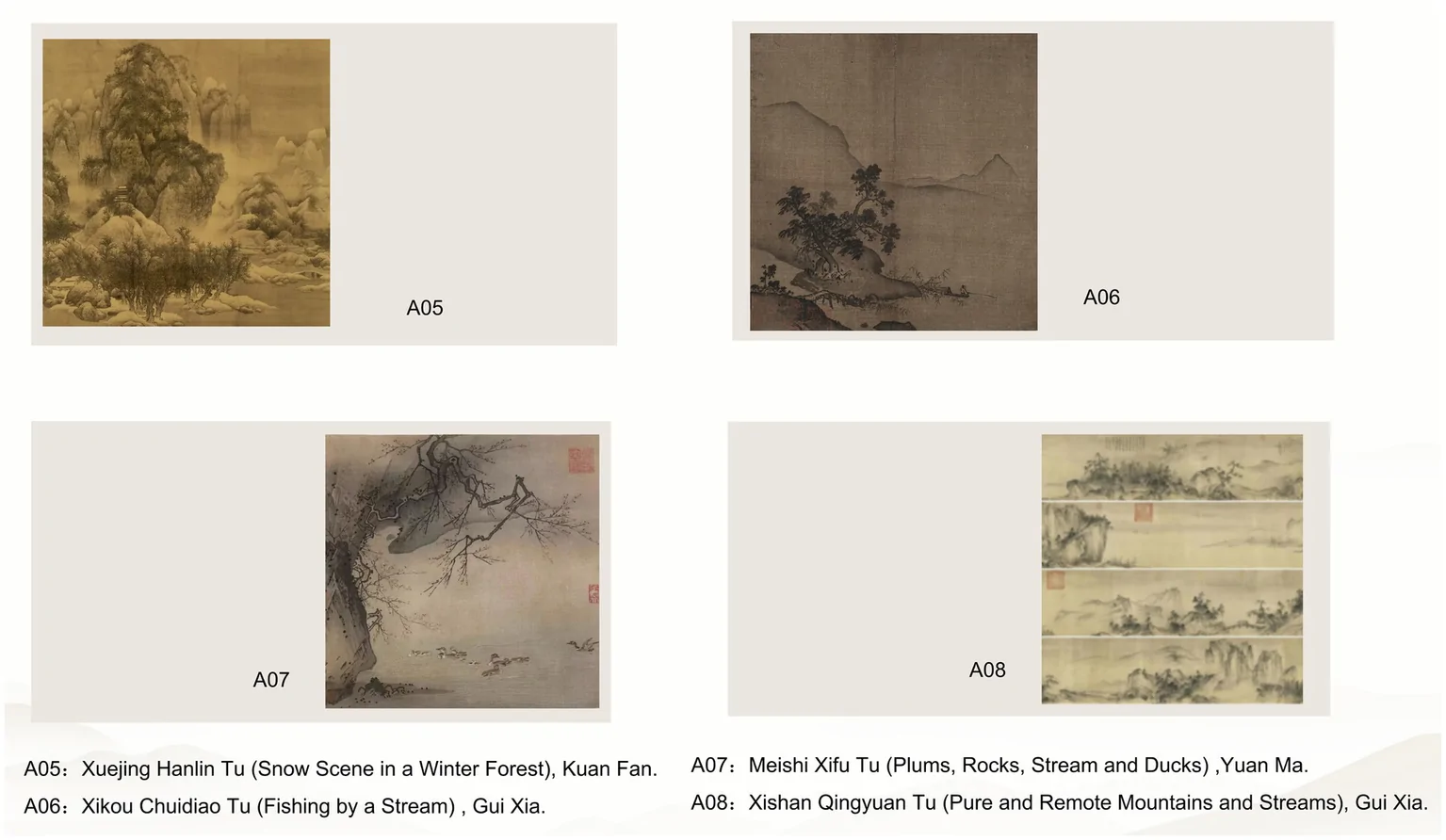
Spatial layering of works.

For example, Xi Guo’s Zaochun Tu (Early Spring), as a paradigmatic Northern Song landscape painting, strictly relies on the “Three Distances” method to build a hierarchical landscape space, employing a tripartite foreground–middleground–background composition to accomplish the spatial depth of the picture. Zaochun Tu (Early Spring), through the massing of objects, density rhythms, the relationship between emptiness and solidity, and the high–low variations in the visual design, forms relatively clear viewing cues that transform the static two-dimensional painting into a three-dimensional spatiotemporal narrative field, thereby perfectly aligning with the narrative logic of spatiotemporal coordination in Song dynasty painting.

The foreground, located at the lower edge of the painting, serves as the starting point of the visual narrative and bears the core functions of grounding and guiding the view, and setting the tonal direction. The left and right sides are occupied by heavy masses of slope rocks and cliffs, with rock contours clear and the rolled-cloud brushwork robust; the depicted objects are finely rendered with a strong sense of mass, establishing a stable visual base for the composition. Between the rocks, shrubs intertwine in a tangled display; withered branches coexist with new shoots, visually echoing the early-spring character of the season. A creek winds out from rock crevices, and the shallow shoreline reveals clearly layered strata. Narratively, the foreground represents a near-field tangible space, dense in imagery, with substantial brushwork and rich detail, providing the viewer with a plausible, palpable footing and a sense of presence. In terms of viewing path, the foreground’s symmetrical, balanced composition yields an inward visual pull; the course of the stream, the arrangement of rocks, and the posture of the trees jointly guide the gaze upward and inward, laying a complete gateway for the middle-ground’s spatial extension and narrative unfoldment. Visually, the foreground is dominated by solidity, with measured density and steady brushwork, establishing a narrative tone across the entire painting that is stable, majestic, and beginning to reveal vitality.

The midground, located in the center of the composition, serves as the core nexus of spatial transformation, gaze traversal, and narrative unfolding, and is the region richest in temporospatial narration. Extending from the foreground, the stream flows upward into the valley interior, with the mountains on both sides opening and facing one another, layering and advancing, their forms embracing to create a deep valley space. A winding boardwalk, a cascading waterfall, and pavilions and towers hidden among the forest—where artificial forms merge with the natural landscape—compose a Song Dynasty landscape narrative scene that is both navigable and inhabitable. Upon entering the midground from the foreground, the progression shifts from a single linear advance to a multi-layered, multi-dimensional wandering state, moving back and forth with the staggered arrangement of the waterfall, boardwalk, valley, and pavilions to form a dynamic touring narrative. In the spatiotemporal narrative dimension, the medium shot achieves a synergistic fusion of spatial depth and temporal dynamics: through progressively layered mountain ranges that construct a space that is simultaneously distant and lofty, and through the dynamic traversal of the gaze, it transforms a static frame into a continuously flowing experience of viewing time, serving as the core carrier of the narrative cadence and visual experience of the entire work’s narrative representation.

The background occupies the upper portion of the frame, centered on the main peak, and represents the ultimate extension of the painting’s space and the culmination of its narrative mood, completing the overall spatial–temporal narrative loop of the image. The central peak rises majestically, with the primary and secondary ridges responding to one another; the mountains ascend layer by layer and extend toward the horizon, maximizing the sense of height and distance to evoke a sublime, expansive, and awe-inspiring landscape. Compared with the foreground’s density and the midground’s richness, the background elements are minimal and the contours refined; rock details are subdued, and broad washes of light ink render the clouds and distant mountains in a seamless blend, yielding a stark contrast between solid and void. From the perspective of spatial construction, the background transcends a concrete navigable space, extending the limited frame’s landscape into an infinite heaven-and-earth space.

Overall, through the hierarchical spatial arrangement of foreground, midground, and background, an orderly spatial-narrative structure is constructed. The spatial levels are interlinked and mutually resonant, breaking the flat attribute of static imagery, enabling the spatial layout itself to constitute a complete narrative syntax and providing the core structural support for the temporal unfolding and content transmission of the visual narrative.

#### 4.3.2 Narrative path: the continuity of narrative unfoldings in visual dynamics

Encoding analysis of narrative nodes and viewing logic reveals that the narrative quality of Song Dynasty painting does not rest on concrete plots, but on the viewer’s visual dynamics, which form a continuous narrative sequence. The composition, through the arrangement of visual elements, the setting of focal points, and spatial turns, designs a viewing path that is fixed yet fluent, relying on a complete visual process of visual fixations, visual transitions, spatial transitions, and viewing termination to achieve an orderly unfolding of the narrative (Figures 4–6).

**Figure 4:**
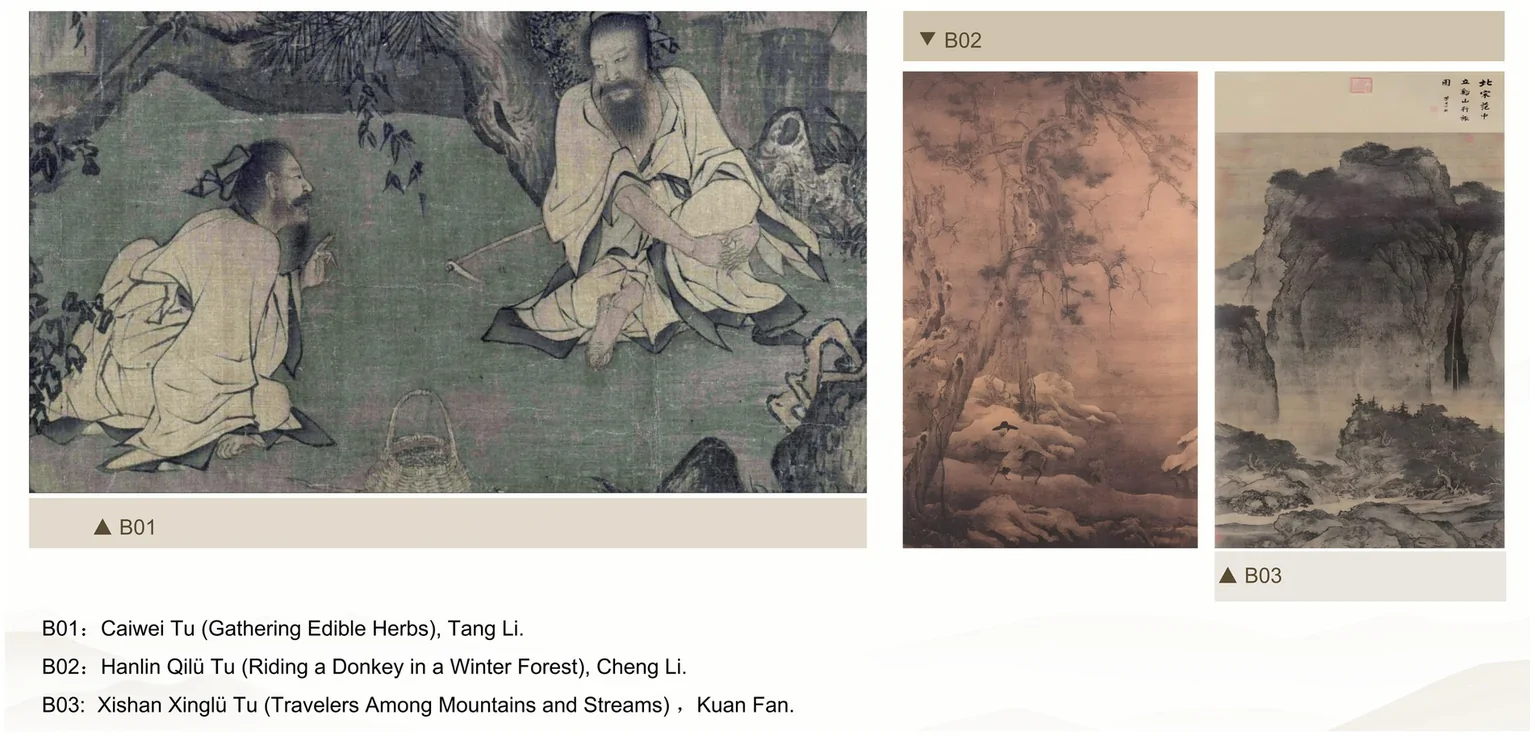
A portfolio of works with a wandering viewing path (1).

**Figure 5:**
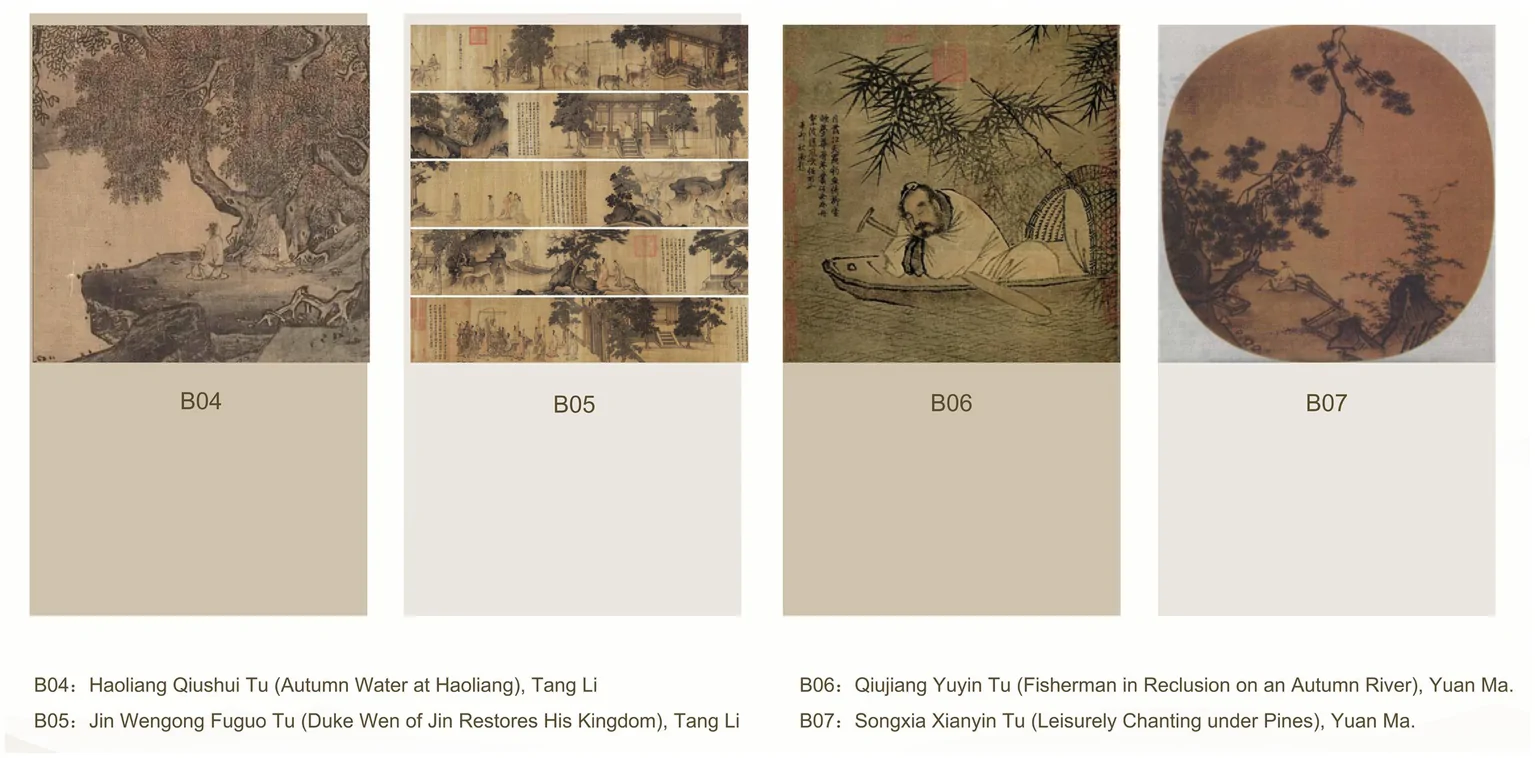
A portfolio of works with a wandering viewing path (2).

**Figure 6:**
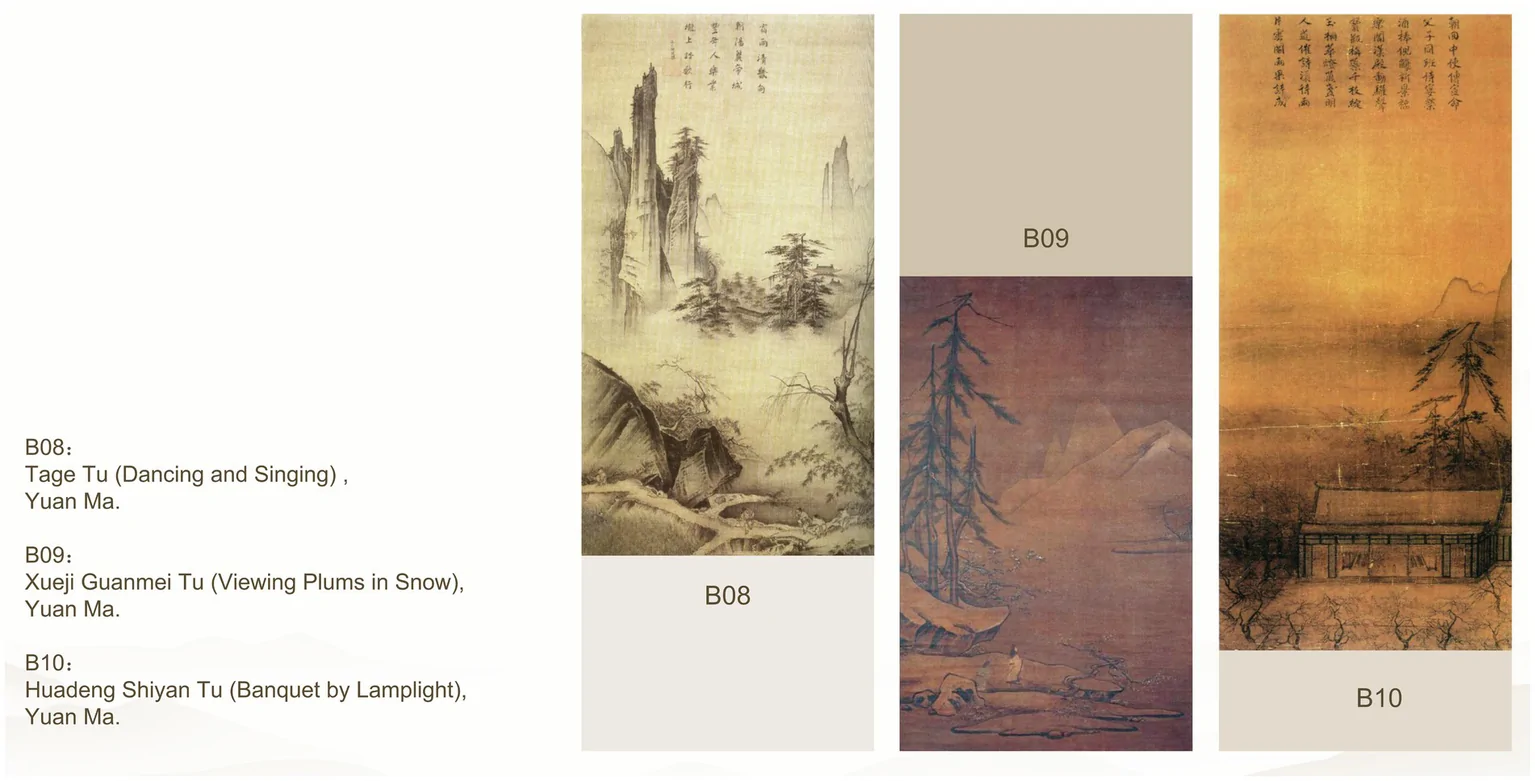
A portfolio of works with a wandering viewing path (3).

Taking Tang Li′s Caiwei Tu (Gathering Edible Herbs) as a case study, the work is a Figure Story Painting; it draws on the historical tale of Boyi and Shuqi refusing Zhou’s porridge and foraging for ferns on Shanyang Mountain. Unlike landscape paintings that guide the eye through near–far hierarchies, this horizontal image constructs a visual migratory path that moves from exterior to interior, from person to scenery, and from action to emotion, through the relationships among figures, the arrangement of objects, and the obstructions within the scene.

The visual focus rests in the painting’s central area, where the two protagonists, Boyi and Shuqi, occupy the visual center, the most important locus of the visual migration. On the right, Bo Yi sits on a rock, body slightly forward-leaning, face oriented toward Shuqi, with a grave expression, as if speaking; on the left, Shuqi squats gathering ferns, body turned toward Bo Yi, holding a fern, listening intently. At this moment, the viewing path no longer progresses unidirectionally but engages in reciprocal drift: the gaze first falls on the more salient Bo Yi, then shifts to Shuqi, and returns between the two, oscillating among the characters’ postures, expressions, and hand gestures. The image uses a smooth, flat rock as a shared platform for the two figures, enclosing them within the same narrative space. Rock outcrops and shade encircle them, forming a relatively enclosed psychological micro-field. At this point, the visual drift has departed from mere spatial exploration and turns to reading the story and the characters’ emotions: the viewer follows the gaze back and forth, experiences the narrative moment of the dialogue, and feels the austere, steadfast spirit. Environment recedes as a backdrop, and the characters’ actions and expressions become the narrative’s core carriers.

Subsequently, the viewing path drifts toward the surrounding environment. Behind Bo Yi, a sturdy tree trunk stands, its thick trunk extending horizontally, with broad leaves forming a natural visual barrier. Rocks and earthen slopes unfold from left to right; the contours of the rocks are rigid, exhibiting Tang Li′s characteristic da fu pi cun (great-axe-cut) texture, while the hillside is overgrown with grasses, composing the wilderness of Shanyang Mountain. The gaze then returns to the center, and the scenery around the central figures begins to simplify: rocks are no longer dense, and the environment shifts from a bustling woodland to a relatively quiet understory clearing. Space transitions from peripheral natural surroundings to a small understory space where the figures reside. The visual drift here undergoes a rhythmic change: the earlier density of objects gives way to simplicity, causing the visual focus to detach progressively from landscape elements and move toward the central figures. The environment ceases to be the subject of viewing and instead functions as a narrative backdrop that foregrounds the characters’ situation.

From this, it is evident that in this type of painting, firstly, by arranging the primary and secondary figures in the composition, employing contrasts between void and solidity, and deploying a sparse-to-dense arrangement to set a core visual focal point, the viewer’s gaze is held within the core narrative region, enabling the reception and reading of the core narrative information and thereby establishing the narrative core. Secondly, visual transition: after the information in the central region has been read, the frame employs visual cues such as line direction, object continuity, and spatial orientation to provide potential viewing paths. The gaze naturally shifts from the central focal point to secondary visual regions, enabling transitions and elaboration of the narrative content and ensuring narrative coherence. Thirdly, spatial progression: the integration of emptiness and fullness across spatial levels and the linking of scenes drive the gaze to dynamically traverse the foreground, middle ground, and background, allowing the narrative to advance with the spatial extension and forming a progressively layered narrative rhythm. Finally, at the end of viewing, the gaze ultimately returns to the background void or the painting’s blank area, completing the overall visual viewing process while realizing narrative closure and the extension of mood, yielding a complete closed-loop narrative experience. This viewing logic, centered on visual dynamics, endows static paintings with temporal properties of sequence, causing fragmented visual elements to form a continuous narrative chain and constituting a distinctive visual-narrative chronology. Consequently, the viewing path in this study is not treated as a directly measurable observer psychology process but as a structural resource for organizing continuous experience and narrative timing within the visual text.

#### 4.3.3 Visual expression: organization of medium characteristics and construction of narrative meaning

Encoding analysis of the visual-expression dimension centers on brushwork, coloration, and the morphology of void and fullness. The results indicate that painting’s visual language is not merely an aesthetic form but the core vehicle that serves spatial narrative, organizes visual experience, and constitutes the construction of narrative meaning. The sample works can be divided into two visual regimes—ink-wash expression and blue-green expression. Through differentiated visual forms, these two regimes construct distinct visual-experience modes, ultimately aiding the generation and expression of the painting’s narrative meaning (Figure 7).

**Figure 7:**
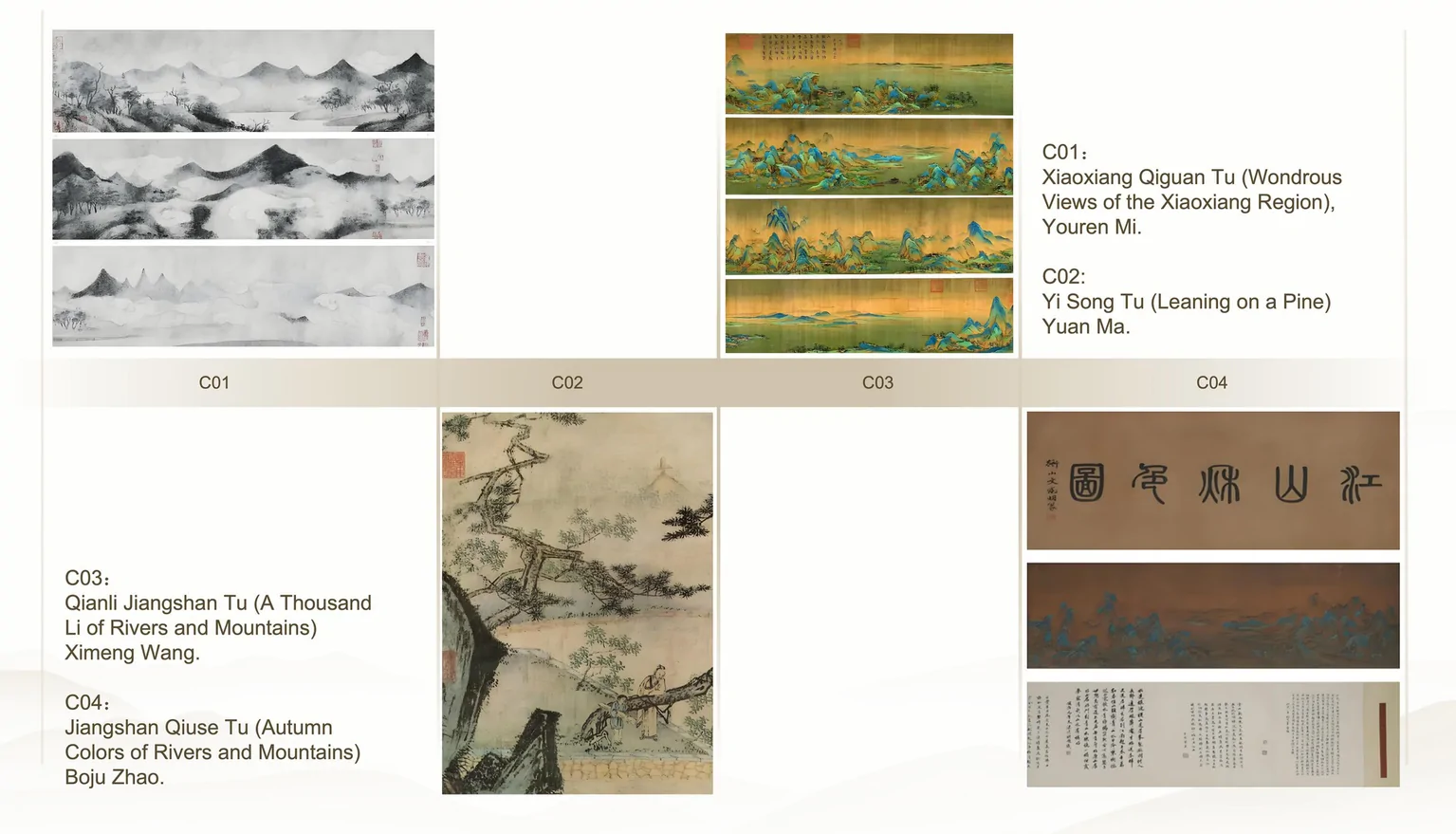
Portfolio of works exhibiting medium-specific forms.

Youren Mi’s Xiaoxiang Qiguan Tu (Wondrous Views of the Xiaoxiang Region) adopts ink-wash as its expressive medium. The composition centers on the modulation of ink density, the contrast between void and solid, and the rhythm of the brushwork. Drawing on a rich spectrum of ink tones to delineate spatial boundaries and to organize spatial order, it orchestrates a flowing, ethereal visual experience. The ink language diminishes the visual impact of literal colors; through the interplay of emptiness and fullness, it fashions spatial qi (spatial aura), offering latent viewing trajectories, and, within a tranquil visual experience, reveals the fluidity and layering of the pictorial space, guided by the rhythm of emptiness and fullness to convey implicit narrative meaning.

The blue-green expression system relies on mineral pigments such as stone-blue and stone-green, applying multiple layers of color to shape stable, cohesive spatial forms through clear color gradations and an orderly visual structure. Explicit color boundaries and object-structure delineate a legible spatial order, producing a regular, solemn visual experience that reinforces the integrity and stability of the narrative space and ensures the clarity of the narrative logic. Ximeng Wang’s Qianli Jiangshan Tu (Wondrous Views of the Xiaoxiang Region) employs gradients of stone-blue, stone-green, and ochre; through pigment layering, glazing, and differential staining, it constructs the spatial hierarchy of mountains and rivers, while color rhythm modulates the visual movement tempo of the long scroll and handscroll, transforming color into a crucial visual medium for constructing narrative space, guiding viewing paths, and rendering the mood.

The two visual regimes differ in form, but their core functions unify, integrating spatial hierarchy with viewing pathways to organize the visual experience. Through differentiated visual-language filtering, sorting, and integration of narrative information, they complete the loop from visual form and experience to narrative meaning, thereby participating in the construction of spatiotemporal co-narration.

#### 4.3.4 Cross-dimensional coordination: the joint organization of space, viewing trajectories, and visual representation

Encoding results further indicate that spatial hierarchy, viewing trajectories, and visual representation are not three independent visual attributes, but form stable synergistic relations within specific narrative units. First, spatial hierarchy provides the structural boundaries of the viewing trajectory; the hierarchical transitions among the foreground, middle ground, and background constitute the basic direction of visual movement. Second, the viewing trajectory further organizes spatial relations into narrative nodes with a sequential order, endowing static space with a continuous temporal structure. Finally, the density of ink, color layering, the contrast between positive and negative space, and the rhythm of brushwork co-occur synchronously with spatial transitions, regulating the visual focal point and the narrative tempo. Consequently, spatial organization provides the structural condition for “where to unfold,” the viewing trajectory forms the narrative sequence of “how to unfold continuously,” and visual representation further determines “with what visual rhythm and form it is presented.” Together, they form a synergistic chain from spatial structure, through path organization, to visual meaning (Table 4).

**Table 4:** Cross-dimensional coordination in presentation.

Synergistic relationships	Primary manifestations	Narrative function
space × viewing path	Foreground, middle ground, and distant background: focal-point transitions	Create a visual sequence
Viewing path × temporal cues	Pausing—transformation—extension	Establish continuity
Space × medium	Form and void; ink tones; color application	Enhance spatial hierarchy
Medium × viewing path	Shifts in color/ink focal points	Modulate visual rhythm
Space × time × medium	Scene, path, and visual rhythm undergo synchronized changes	Create spatiotemporally coordinated narration

### 4.4 Stable narrative mode and cross-dimensional coordinated features

An integrated coding of sample works and cross-dimensional cooperative analysis reveals that Song dynasty painting, despite differences in subject matter, composition, and visual language, exhibits relatively stable coordination among spatial organization, temporal cues, narrative nodes, viewing routes, and visual media. Spatial hierarchies provide the structural field for narrative development; narrative nodes and temporal cues form a sequence; viewing routes connect different nodes; ink density, coloring, and other media features such as tonality and density or sparseness participate in organizing visual focal points and rhythm. Accordingly, spatiotemporal coordination is not an attribute of a single visual element, but a relational structure formed by the joint contribution of multiple visual cues (Figure 8). Further analysis indicates that spatial organization and narrative nodes together constitute the narrative skeleton. Foreground, middle ground, and background, as spatial layers, provide positional relations for figures, architecture, mountains, and other key imagery, and through spatial transitions establish structural connections between scenes; narrative nodes, temporal cues, and viewing routes collectively form a narrative sequence that renders latent temporal order among scenes, actions, and visual focal points within static images. The viewing route referred to here is an analytical reconstruction based on spatial relations within the frame, visual salience, and narrative nodes, and does not represent actual viewer eye-tracking trajectories. At the same time, visual-media features participate in organizing spatial and narrative rhythm. Form features such as ink density, coloring, solidity/void, and sparse/dense arrangement, in combination with spatial hierarchies and narrative nodes, participate in configuring visual focal points, transitions, and rhythm, thereby strengthening connections among different narrative units. In sum, Song dynasty painting exhibits a relatively stable narrative mode of “spatial construction—node concatenation—route expansion—media organization,” namely, space establishes the narrative field, nodes and temporal cues form a sequence, viewing routes connect the visual unfolding, and media features organize focal points and rhythm. This mode indicates that the narrative quality of traditional painting does not originate from a single visual element, but rather manifests as the coordinated organization of multi-dimensional visual cues, providing a structured pathway for conducting systematic narrative analysis of painting as a non-linguistic visual text.

**Figure 8:**
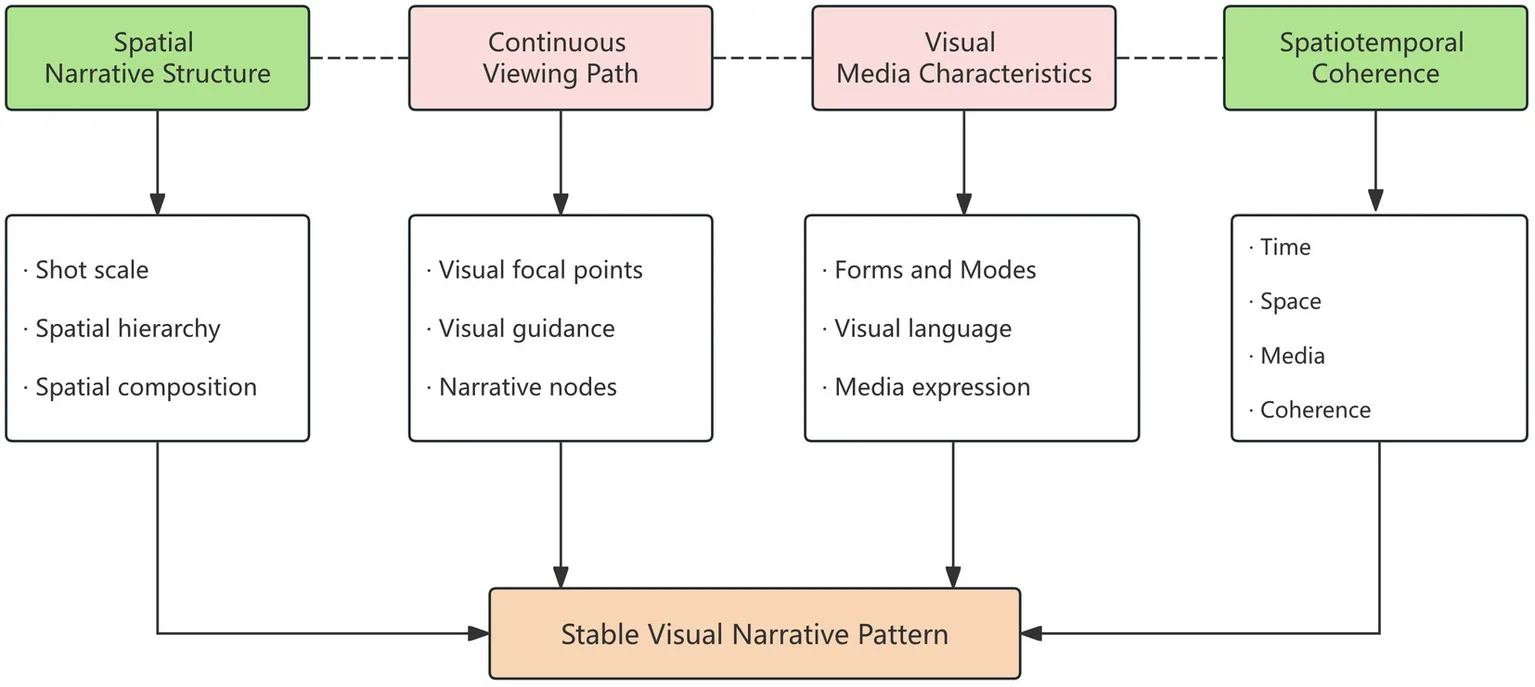
Visual narrative logic. Space constructs the narrative field, nodes and time from the sequence, the viewing path connects the unfolding, and media features organize the focus and rhythm.

## 5 Discussion

### 5.1 Spatiotemporal coordination in song dynasty painting reflects the organization of visual narrative

This study, based on a textual analysis method, systematically codes traditional visual narratives. The results indicate that the spatial organization pattern, the setting of narrative nodes, and visual presentation strategies jointly constitute the spatiotemporal co-narrative structure of the painting. This finding suggests that existing narrative texts (Yang, 2022) do not rely solely on the image to reproduce natural landscapes, but rather organize the viewer’s reading process through spatial relations (Intxaurbe et al., 2024), viewing paths (Chamberlain et al., 2021), and visual hierarchies (Yao et al., 2022), enabling static images to exhibit continuous narrative features.

This result resonates with the visual narrative research view that “images can organize experience” (Schoch and Ostermann, 2025). Bruner proposed that the essential function of narrative is to integrate discrete experiences into a meaningful whole (Bruner, 1986); Polkinghorne further argued that the continuity of experience derives from the organization of temporal relations by narrative, rather than relying solely on events themselves (Polkinghorne, 2010). When applied to Song Dynasty painting, it becomes evident that spatial layout not only fulfills visual organizational functions but also serves as an important medium for narrative progression. Whether it is the continuous spatial hierarchy formed by Northern Song panoramic compositions, or the localized focal points and negative-space organization formed by Southern Song cornered compositions, both demonstrate a cooperative relationship between the painting’s space and the viewing path. This implies that space in traditional painting is not merely physical space but a structural resource for organizing the unfolding of narrative (Vodyanyk et al., 2026). This finding indicates that spatial organization is not only a formal feature of traditional painting but also an important structural unit in the unfolding of visual narrative. By jointly organizing the viewing process through spatial hierarchy, negative-space allocation, and scene transformation, static images can form a continuous narrative text, providing a new theoretical entry point for the analysis of narrative methods in traditional visual texts (Ma, 2021). Moreover, this study does not interpret different compositional modes as causal sources of the viewer’s psychological state; rather, it understands them as distinct strategies for organizing visual narrative. Different spatial structures correspond to different viewing paths, which in turn generate different modes of experiential organization. This interpretation avoids inferring psychological mechanisms directly from the results of textual analysis and keeps the study’s conclusions within the evidentiary scope that narrative analysis can support. The viewing paths and visual experiences identified in this study are analytic representations at the level of the visual text, rather than actual eye-movement trajectories or psychological responses of observers. Consequently, this study reveals that Song Dynasty paintings, in their visual organizational structure, provide narrative paths that are directional, continuous, and hierarchical, and these structures can be understood within a narrative psychology framework as visual forms of experiential organization and meaning construction.

### 5.2 From visual imagery to narrative text: textualization pathways in traditional painting

Narrative research emphasizes that the objects of study include not only linguistic texts but also multimodal texts capable of organizing experience (Gubman and Anufrieva, 2024). Herman notes that as long as an image can form a temporally connected event structure, it can be analyzed as a narrative text (Herman, 2009). However, in traditional painting studies, most research still predominantly centers on formal language, iconography, or cultural meaning, with relatively little analysis treating painting as an integrated narrative text with intrinsic organizational logic. This study seeks to address this methodological question by converting Song dynasty paintings into analyzable visual narrative texts (Cohn and Magliano, 2020) and by constructing an encoding framework composed of spatial hierarchies, viewing paths, and visual-media representations. Compared with traditional painting research that emphasizes a work’s style, painter case studies, and cultural background, this study focuses more on the organizational relationships among internal visual elements, treating spatial levels, viewing paths, character activity, and visual-media representations as the fundamental units jointly constituting the narrative process (Fraser and Fraser, 2024). This analytic approach renders painting research, previously relying on experiential interpretation, into a more transparent workflow, enhancing the systematicity and traceability of text analysis (Brown and Collins, 2021).

At the same time, the results indicate that traditional painting, although lacking linguistic narration, can still form a continuous narrative process through spatial transposition, visual focus, and scene organization. Visual imagery and linguistic text differ in their expressive media, yet both can organize experience and construct meaning through narrative structure (Feder, 2021). This study further demonstrates that viewing paths constitute an important structural condition for the sequence of visual exploration within a frame (Chamberlain et al., 2021), and they play a crucial role in linking spatial organization with narrative progression. Treating viewing paths as a vital component of visual narrative helps transcend the limitations of traditional image analysis, which tends to emphasize static composition, and further broadens the understanding of how narrative methods organize visual experience (Moliako and Shepelova, 2026). Consequently, viewing traditional painting as a visual narrative text not only broadens the scope of narrative analysis but also provides a new analytical trajectory for visual arts research. Methodologically, the value of this study lies not in merely translating traditional painting into language text, but in demonstrating that visual texts can establish traceable narrative-analytic units based on their own spatial, temporal, and viewing organization.

### 5.3 Implications, psychological relevance, and scope of inference of narrative-analytic research

The core contribution of this study is not to reassess the significance of Song Dynasty painting within art history, but to test and extend the applicability of narrative-analytic methods to nonlinguistic visual texts (Grant et al., 2026). First, this study integrates visual-text analysis (Brown and Collins, 2021) with narrative analysis (De Fina, 2021), constructing a structured analytical framework suitable for traditional painting. The framework integrates spatial organization (Davidson, 2023), temporal cues, viewing paths (Chamberlain et al., 2021), and narrative nodes into a unified analytic system, providing an actionable analytical workflow for visual narrative research and addressing the lack of tools for narrative analysis in traditional painting. Second, this study extends the applicability boundaries of narrative research methods (de Medeiros and O’Neill, 2022). Existing narrative analysis has primarily concentrated on interviews, life histories, literary texts, and digital media, whereas this study likewise treats traditional painting as a target for narrative analysis. By converting static images into structured narrative texts, narrative-analysis methods can enter the field of visual arts research and offer a shared analytic language for visual expressions across different cultural traditions (Yi et al., 2025). Finally, the study provides cases from Chinese traditional art for cross-cultural visual-narrative research (Lovell and Wallace, 2019). International visual-narrative research has long centered on Western modern media, with relatively limited attention to Eastern traditional painting. The spatial organization manifested in Song Dynasty painting (Chamberlain et al., 2021), and temporal unfolding exhibit distinctive cultural features, and likewise suggest that while the manifestations of visual narrative differ across cultural backgrounds, they can all be organized through narrative structure to experience and construct meaning. Consequently, Visual presentation strategies not only shape the artwork’s artistic style but also participate in the generation of meaning in visual narrative (Yang, 2022). Different visual modalities, by influencing narrative tempo, visual focus, and the organization of experience, collectively constitute a major component of the construction of meaning in visual texts, further enriching the analytical dimensions of narrative methods for nonlinguistic narrative resources, and contributing to the cross-cultural broadening of narrative research, as well as extending the range of narrative-method applications in non-Western visual cultures.

From a psychological perspective, the contribution of this study lies primarily in the narrative dimension of experience organization, rather than in direct measurement of the psychological states of historical figures or of actual observers. Narrative psychology emphasizes how experiences are organized into wholes that possess continuity and meaningful connections; this study extends this issue to nonverbal visual texts, examining how within a painting the “presented experience” is organized through spatial hierarchy, gaze paths, temporal cues, and visual presentation. Therefore, what this study discusses is the organization of experience and the structure of meaning in visual texts, rather than the historical painter’s real psychological state, nor empirical measurement of contemporary viewers’ actual viewing behavior. This distinction grounds the psychological significance of the study in the analysis of narrative structure and experiential representation, rather than in inferences about underlying psychological mechanisms.

### 5.4 Limitations and future research

Although this study yields important findings regarding the spatiotemporal co-narrative structure formed by the spatial organizational patterns, the setting of narrative nodes, and the visual-presentation strategies of Song dynasty paintings, there remain certain limitations. First, this study employs a textual-analysis approach, focusing on the internal narrative structure of the paintings; as a result, the conclusions principally reflect the organizational characteristics of the visual text and cannot directly infer viewers’ actual psychological responses or cognitive processes. Future work could triangulate these visual-narrative analysis results with methods such as eye-tracking experiments, interviews, or records of the viewing process to provide further validation. Second, the study analyzes primarily representative Song dynasty paintings; while this choice highlights important features of Song painting, it has not encompassed all developmental stages of traditional Chinese painting. Future research could further compare the continuity and differences in narrative organization between Yuan, Ming, and Qing dynasty paintings and Song dynasty paintings, enriching the historical dimension of traditional painting visual-narrative research. Third, the proposed visual-narrative coding framework needs testing across a range of media. Future work could apply it to calligraphy, murals, prints, and digital art to continually refine a method for multimodal texts and provide additional support for cross-cultural visual narrative research. Therefore, this study offers a textual interpretation of visual-narrative structure and experiential organization, not empirical measurements of viewers’ responses.

## 6 Conclusion

This study treats Song Dynasty painting as its object of study and, within the frameworks of narrative psychology and visual-narrative theory, employs a textual analysis method to systematically code and conduct narrative analysis on representative works. The findings indicate that in Song Dynasty painting, spatial hierarchy, viewing paths, and the representation of visual media collectively constitute a spatiotemporal visual-narrative structure. The spatial layout, viewing paths, and temporal cues within the paintings are not independent; rather, they participate in an interconnected organizational process that yields a continuous narrative logic, causing static images to exhibit temporally extended visual-textual characteristics. At the level of visual-textual analysis, this suggests that traditional painting is not merely an artistic form of expression but can be studied as a visual-narrative practice that organizes presented experience and constructs narrative meaning.

Methodologically, this study repositions Song Dynasty painting within a visual-narrative analysis framework, constructing a spatiotemporal co-narrative analysis framework centered on spatial organization, temporal cues, narrative nodes, and viewing pathways, converting relatively dispersed visual forms in traditional painting into narrative units with clearly defined analytical boundaries. This approach transcends traditional analyses that rely solely on formal style or cultural symbols, offering a relatively transparent, systematic, and traceable narrative-analytic path for nonlinguistic visual texts and expanding the applicability of narrative methods in the study of traditional visual arts.

The study shifts the focus from what painting presents to how visual texts organize experience and generate meaning. This perspective indicates that narrative is not limited to the linear text of language, characters, and events, but can also be realized through spatial relations, viewing pathways, temporal cues, and visual transitions to yield structured expression. Therefore, Song Dynasty painting provides a culturally specific analytical case for examining the organization of experience and meaning construction in nonlinguistic texts and offers new research directions for applying narrative methods in Eastern traditional visual arts and cross-cultural visual studies. It should be emphasized that the narrative structures and experiential organization discussed herein pertain to the visual-text level and do not entail inferences about the historical painter’s actual mental states, nor do they make causal judgments about observers’ actual psychological responses or cognitive mechanisms. Future research could test the applicability of the proposed framework to other visual arts, cross-cultural texts, and multimodal materials, and, combining empirical methods like viewing experiments, eye-tracking, and interviews, validate the relationship between visual-narrative structure and viewing experience.

## Data Availability

The original contributions presented in the study are included in the article/supplementary material, further inquiries can be directed to the corresponding author.
